# Preference of acromegaly patients for treatment attributes in Spain

**DOI:** 10.1007/s12020-023-03462-z

**Published:** 2023-07-28

**Authors:** Carmen Fajardo, Cristina Álvarez-Escola, Betina Biagetti, Rogelio Garcia-Centeno, Raquel Ciriza, Laura Sánchez-Cenizo, Marcos Díaz-Muñoz

**Affiliations:** 1 Endocrinology Department, La Ribera University Hospital, Alzira, Valencia Spain; 2 Endocrinology Department, La Paz University Hospital, Madrid, Spain; 3 Diabetes and Metabolism Research Unit, Vall d’Hebron University Hospital and Vall d’Hebron Research Institute (VHIR), Universidad Autónoma de Barcelona, Barcelona, Spain; 4 Endocrinology Department, Gregorio Marañon University Hospital, Madrid, Spain; 5 Spanish Association of People Affected by Acromegaly (Asociación de pacientes Afectados por Acromegalia), Huesca, Spain; 6 Medical Affairs Department, Pfizer S.L.U, Alcobendas, Madrid, Spain

**Keywords:** Acromegaly, Growth hormone, Quality of life, Treatment, Preferences

## Abstract

**Objective:**

Acromegaly is a rare disease caused by increased growth hormone secretion and a subsequent increase in insulin-like growth factor I (IGF-I) levels. Patients display multiple comorbidities that affect their quality of life (QoL). Treatment aims to maintain good biochemical control, tumour control and reduce the risk of comorbidities; however, their impact on QoL has been overlooked until recently. We interviewed patients to explore their preferences with regard to treatment attributes.

**Design:**

A cross-sectional study based on interviews and a discrete choice experiment (DCE) in a Spanish cohort.

**Methods:**

Adult patients diagnosed with acromegaly ≥1 year before the start of the study and under treatment were included. Treatment attributes were collected from patient testimony during face-to-face interviews. Then, a DCE was performed to elicit patient preferences for certain treatment attributes.

**Results:**

Sixty-seven patients completed the study. QoL improvement was the most important treatment attribute (37%), followed by IGF-I control (20%), blood sugar control (17%) and tumour control (13%). Secondary attributes were pain associated with the route of administration (7%), diarrhoea (2%), administration method (2%) and storage conditions (2%). We then calculated the theoretical share of preference for existing treatments, based on the individual preference utility for each attribute and level. Pegvisomant obtained the highest share of preference overall, and the highest preference as a second-line treatment (53 and 95%, respectively).

**Conclusions:**

QoL greatly influences patient treatment preference. Since acromegaly patients are informed and aware of their disease, treatment choices should always be shared with patients.

## Introduction

Acromegaly is a slowly progressive disease caused by an increase in growth hormone (GH) secretion, mainly due to a pituitary adenoma, with a consequent increase in insulin-like growth factor I (IGF-I) [1–3].

It is a rare disease, with an estimated prevalence of 60 cases per million worldwide, although some studies have shown that this could be underestimated [4]. Since the onset of the pathology is difficult to determine due to the lack of early pathognomonic signs, diagnosis is often delayed by between 2-10 years. In terms of the distribution of the illness, both sexes are evenly affected [4].

Acromegaly is associated with multiple comorbidities, premature mortality, and physical disfigurement [1–3]. The most serious consequences include type 2 diabetes, hypertension, increased risk of cardiovascular disease, risk of developing cancer, arthropathy and sleep apnoea [1–3]. In fact, the presence of diabetes mellitus, cardiovascular disease and hypertension at the diagnosis is significantly associated with reduced survival [5].

Acromegaly patients experience decreased energy and psychological disturbances (loss of initiative, mood lability, low self-esteem, depression and anxiety) that significantly affect their quality of life (QoL) [3, 6–8].

Taking acromegaly treatment into consideration, patients usually undergo surgery to remove the primary pituitary tumour. When surgery is not definitive, first-generation somatostatin receptor ligands (1G-SRL, lanreotide, or octreotide) are considered the first line of pharmacological treatment. When SRLs provide insufficient biochemical control of the disease, treatment should be complemented or switched to pegvisomant, a GH receptor antagonist, or to pasireotide, a second-generation SRL. Radiotherapy, especially stereotactic radiotherapy, may be used in case of residual tumour mass following surgery, and if pharmacological therapy is unsuccessful or not tolerated [9, 10].

Lanreotide and octreotide are available as monthly injections. According to the meta-analysis performed by Carmichael et al., 55% of patients taking 1G-SRL may normalise IGF-I [11]. 1G-SRL may also reduce tumour mass in 52% of cases [12]. Moreover, reported 1G-SRL analogue effects on blood sugar can be negative, positive, or neutral [13]. The main reported adverse effect was diarrhoea and injection-side reactions. Both lanreotide and octreotide should be stored in a refrigerator [14, 15].

Pegvisomant is prescribed as a second-line pharmacological treatment when patients are not controlled with 1G-SRLs or do not tolerate the treatment [16]. It is administered daily by subcutaneous injection, and can normalise IGF-I in 92% of cases in phase 3 clinical trial [16, 17] and 65% [18] to 74% [19] in clinical practice. Pegvisomant has a neutral effect on tumour size, and beneficial effects on blood sugar metabolism (reduction in fasting insulin, fasting blood sugar, HbA1c and increased insulin sensitivity) [16, 19]. The main reported adverse events are headache, diarrhoea and injection-site reactions. The drug can be stored at room temperature [16].

2G-SRL, pasireotide, is an intramuscular monthly injection that may control 25–26% of patients not responding to 1G-SRLs. It has been shown to reduce the tumour mass in 19% of patients that do not respond to 1G-SRLS [20]. With pasireotide, hyperglycaemia is reported as an adverse event in 31–67% of cases and diabetes in 21–26%; approximately 48% of patients in one study required the start of new antidiabetic medication [20–23]. Pasireotide must be stored refrigerated and should be administered by a healthcare professional [23].

The goals of acromegaly treatment are overall long-term biochemical control, control of tumour mass and reduced risk of developing systemic comorbidities, thereby reducing mortality.

In recent years clinicians have increasingly focussed on patient perception of mental and physical health, and numerous questionnaires such as PASQ or ACROQoL have been developed and validated [24, 25]. Clinical data, such as GH and IGF-I levels, do not always correlate to patient-perceived health [26], and the development of ACRODAT^®27^ (Acromegaly Disease Activity Tool), a specific tool for measuring acromegaly disease activity, improved understanding of the condition by including QoL among the parameters evaluated. However, the perception of QoL is not the same for patients and healthcare professionals [28]. In general, although it improves after therapy, patients endure suboptimal QoL due to previously unresolved issues caused by the disease [29]. QoL is highly compromised in acromegaly patients, and the psychological burden is especially heavy (morphological changes are among the factors most frequently reported). These patients are willing to learn about their disease and collaborate with clinicians to minimise the impact of acromegaly on their life [29]. In spite of evidence from studies such as AcroVoice [3] that highlight the beneficial effect of patient-centred parameters on disease activity, Marazuela et al. recently showed that IGF-I and tumour mass control are still the main drivers of treatment change, and that patient-centred parameters such as QoL or symptoms were not always considered [30].

Evidence of patient opinions about their disease is scarce. In 2018, a Spanish expert consensus issued the recommendation that QoL should be systematically assessed. However, its importance in relation to therapeutic decisions was not established [31]. Nevertheless, the authors agreed on the importance of post-surgical diagnosis and an acromegaly-centred management approach to determine a patient’s QoL [31]. Our aim was to gain further insight into the patient’s perspective of their disease, unmet needs and treatment preferences.

## Material and methods

To elicit patient preferences for treatment, this study was carried out in two phases: (1) a qualitative phase that included patient group interviews to define the attributes and levels of acromegaly drugs that are relevant to them; (2) a quantitative phase, in which patient preferences were elicited using a discrete choice experiment (DCE) approach. This study followed the code of conduct of the Ethics Committee for Medical Research of the Community of Madrid (*Comité de Ética de la Investigación con Medicamentos*) (Approved May 26 2020, code: CEP130900).

### Patient recruitment

For the qualitative part of the study, patients were recruited by the Spanish Association of People Affected by Acromegaly (http://tengoacromegalia.es/ Tengo Acromegalia, Asociación Española de Afectados por Acromegalia). For the DCE study, they also distributed access to the questionnaire among members. Access to the questionnaire was also given by study investigators to their acromegalic patients.

Although patients were aware that the study was funded by a pharmaceutical company, the identity of the study sponsor was hidden from patients throughout the study to avoid bias. Patients did not have any contact with any employee of the sponsor at any time before or during the fieldwork.

### Inclusion/exclusion criteria

Adult patients with acromegaly were eligible to enter the study provided they had been diagnosed at least 12 months before completing the questionnaire, were under treatment at the time of the study, and were willing to sign the informed consent form and complete the online questionnaire. Patients who had been involved in a market study on acromegaly in the previous month were excluded. Patients affiliated with a pharmaceutical laboratory, governmental regulatory agency, market study agency or advertising agency were also excluded.

### Sample size calculation

Based on acromegaly prevalence (60 cases per million people) [4] and the current Spanish population (47.62 million people) [32], it was estimated that there are around 3000 acromegaly patients in Spain. Assuming a ±10% error, a minimum of 93 participants were required. This calculation was performed for a finite population, with a confidence level of 95.5% and unfavourable sampling conditions of *p* = *q* = 0.50. A rule of thumb for conjoint analysis sampling [33] of NxTxA/C (N=no questionnaires; T=No tasks; A=No alternatives; C=Max. No attribute levels) was applied to check the appropriateness of the sample size for the DCE. It was estimated at 93 × 14 × 2/3 = 868, which is higher than the established threshold of 500.

### Qualitative phase

Two face-to-face group interviews lasting 2 h each and involving four and five adult post-surgical patients, respectively, took place in Madrid and Barcelona in September 2019. The group was chaired by an experienced moderator, belonging to an independent market research agency (Adhara Marketing, Madrid)

To explore participant opinions and personal experiences with the pathology, patient testimony was collected, and treatment variables and attributes for injectable treatments considered important by the participants were then defined. Finally, the participants ranked the attributes according to their importance.

Using Bayesian hierarchical modelling, the percentage of levels and attributes were converted into utilities. Utilities are the units that reflect the value of the satisfaction/happiness of the patients.

### Quantitative phase

#### DCE design

Attributes and levels defined on the basis of on aforementioned qualitative interviews are shown in Table 1. The questionnaire items were validated by expert endocrinologists. The relevance of the attribute for patients was rated, and levels were described. The levels of each attribute corresponded to an existing treatment, if possible. Supplementary Table 1 summarises the correspondence of each level to an existing treatment. To estimate patient preference, octreotide and lanreotide treatment were unified; however, it is important to note that octreotide can only be injected by a primary care/hospital nurse.

**Table 1:** Description of levels and attributes

Levels	Attribute 1: IGF-I level control
**1**	IGF-I levels control in 9 out of 10 patients
**2**	IGF-I levels control in 6 out of 10 patients
**3**	IGF-I level control in 3 out of 10 patients
**Levels**	**Attribute 2: Tumour control**
**1**	Reduces tumour size
**2**	Does not reduce tumour size
**Levels**	**Attribute 3: Administration methods**
**1**	Daily self-administered injection at home
**2**	Monthly self-administered or nurse-administered injection in primary care/hospital
**3**	Monthly nurse-administered injection in primary care/hospital
**Levels**	**Attribute 4: Pain associated with the method of administration**
**1**	Minimal injection pain and no redness/ bruising/ skin hardening
**2**	Minimal injection pain but may cause redness/ bruising/ skin hardening
**3**	Injection may be painful and cause redness/ bruising/ skin hardening
**Levels**	**Attribute 5: Adverse events: diarrhoea**
**1**	Diarrhoea in 1 out of 10 patients
**2**	Diarrhoea in 3 out of 10 patients
**3**	Diarrhoea in 4 out of 10 patients
**Levels**	**Attribute 6: Blood sugar control**
**1**	Improved blood sugar (or diabetes) or blood sugar medication down-dosed
**2**	Blood sugar (or diabetes) or blood sugar medication unaffected
**3**	Blood sugar higher (or diabetes onset), or blood sugar medication may be required/up-dosed
**Levels**	**Attribute 7: Storage conditions**
**1**	Storage at room temperature
**2**	Cold storage (fridge)
**Levels**	**Attribute 8: Quality of life**
**1**	Improves quality of life
**2**	Does not improve quality of life

*IGF-I* insulin-like growth factor I

The questionnaire was tested on three members of the Spanish Association of People Affected by Acromegaly to assess its understandability. The questionnaire was self-administered without the intermediation of the investigators or the patient advocacy group.

Choice tasks were designed by creating task profiles that were as statistically independent as possible, with equally balanced two-way frequency of level combinations, meaning that each attribute level was evaluated an equal number of times as all levels of the other attributes (for example, attribute 1, level 1, was presented the same number of times as attribute 2, level 1).

A total of eight attributes, with a maximum of three levels, were combined and reduced using an orthogonal design for optimal level rotation to generate 14 pairs of two-option choices, which entailed 938 discrete choices of treatment. 'No treatment' was not included as an option. By selecting options, respondents indirectly rated the relative value of a particular attribute over another (Table 2). The scenarios/combinations presented do not necessarily represent any existing treatment, even though each attribute and level are derived from existing injectable treatments.

**Table 2:** Sample of discrete choice task

	Treatment A	Treatment B
**IGF-I level control**	IGF-I level control in 3 out of 10 patients	IGF-I level control in 9 out of 10 patients
**Tumour control**	Reduces tumour size	Does not reduce tumour size
**Administration methods**	Daily self-administered injection at home	Monthly self-administered or nurse-administered in primary care/hospital
**Pain associated with the administration method**	Injection may be painful, cause redness/ bruising/ skin hardening	Minimal injection pain and no redness/ bruising/ skin hardening
**Adverse events: diarrhoea**	Diarrhoea in 1 out of 10 patients	Diarrhoea in 4 out of 10 patients
**Blood sugar control**	Blood sugar higher (or diabetes onset) or blood sugar medication may be required/up-dosed	Improved blood sugar levels (or diabetes) or blood sugar medication down-dosed
**Storage conditions**	Storage at room temperature	Cold storage (fridge)
**Quality of life**	Improves quality of life	Does not improve quality of life

In each task, the participant has to choose between two alternatives of treatment that are described by eight attributes. Each attribute assumes a series of levels. The specific combination of levels for the given attributes defines a unique treatment alternative

*IGF-I* insulin-like growth factor I

#### Questionnaires

Data collection took place between August 2020 and March 2021. The methodology employed was the DCE. Questionnaires were self-administered by the patient using a Computer Assisted Web Interviewing (CAWI) model. Following an *ad* *hoc* questionnaire designed for classification and dimensioning, participants were presented with 14 scenarios and were asked to choose between two exclusive options in each scenario. The maximum duration of the task was 10 min.

Subanalyses were performed on subpopulations, according to the number of years since diagnosis, presence of diabetes, and line of treatment.

### Statistical analysis

#### DCE analysis

Hierarchical Bayesian modelling was used to estimate utilities for each attribute level, and the relative importance of attributes was therefore determined as choice predictors. Logistic coefficients at the level of the individual respondent for each of the attributes and levels were extracted from the grid of options used to construct the discrete choice task profiles. This analysis provided the basis for all further analyses of the choice data. Participant preferences were estimated at individual and aggregate levels.

The paradigm behind the design was a part-worth model in which the “worth” of a product profile comprised the sum of the individual 'worth' of each of the attribute levels (part worth). Part-worth utilities were derived from the task design information and the 14 task choices completed by each respondent using Hierarchical Bayesian modelling.

#### Raw utilities

Utilities measure the value of each attribute level to the respondents. Raw utilities were used to scale the data: the sum of utilities for each attribute level and each respondent equals zero.

#### Relative importance

Relative importance (RI, the degree to which changes across the levels of an attribute will have an impact on the share of preference) was calculated as individual and aggregate relative importance:

1. Individual RI (IRI): utility range (maximum minus minimum) of each attribute was calculated for each participant and summed. The attribute proportion of that sum is its IRI.
2. Aggregate relative importance (ARI): utilities for each level of each attribute were averaged across participants, and then range-sum-average calculation was applied to the averages.

ARI sums to 100% across attributes with IRI calculated relative to ARI.

#### Software

The package ‘ChoiceModelR’ was utilised to assess coefficients of a Hierarchical Bayes Multinomial Logit Model. It implements an MCMC algorithm to estimate a hierarchical multinomial logit model with a normal heterogeneity distribution. The algorithm uses a hybrid Gibbs Sampler with a random walk metropolis step for the MNL coefficients for each unit.

## Results

### Respondent sociodemographic and clinical characteristics

**Fig. 1:**
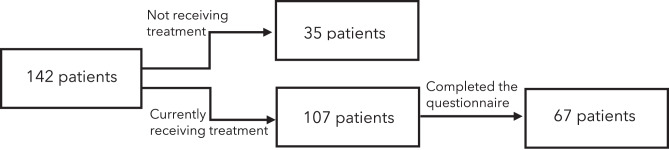
Quantitative phase, patient recruitment flowchart

One-hundred-and-forty-two patients were initially invited to take part in the study; however, 35 were no longer on treatment, and were therefore excluded. Of the remaining 107 patients, 67 completed the questionnaire (Fig. 1), with an a posteriori error of ±11.8%. This still resulted in a NxTxA/C of 625, which is still higher than the 500 threshold. Table 3B summarises patient demographics and clinical characteristics.

**Table 3:** Participant sociodemographics and clinical characteristics

	Respondents (Conjoint Analysis) (*n* = 67)
**SEX**	
Women	63%
Men	37%
**AGE**	
Mean, years old (SD)	50 (10.4)
18–44	42%
45–54	42%
≥55	16%
**EDUCATIONAL LEVEL**	
Primary school not completed *n*(%)	2 (3)
Primary school	22 (33)
Secondary school	16 (24)
University or higher	27 (40)
**PREVIOUS INJECTABLE TREATMENTS**	
Yes	61%
No	39%
Not sure	—
**CONCOMITANT DISEASES**	
Mean, *n* (SD)	3.3 (2.0)
Joint pain	64%
Cholesterol	39%
Pituitary hormone deficiency (TSH, testosterone, etc.)	39%
Diabetes or high blood sugar	31%
Hypertension	31%
Carpal tunnel syndrome	28%
Sleep apnoea	27%
Colorectal polyps	24%
Thyroid nodules (goitre)	22%
Heart diseases	15%
None	6%
**YEARS SINCE DIAGNOSIS**	
Mean, years (SD)	11.4 (8.0)
≤3	21%
4–8	30%
9–15	25%
≥15	24%
**PRIOR SURGERY**	
Yes	91%
*Complete resection, n*	61
Yes	12%
No	75%
Not sure	13%
No	9%
**PRIOR RADIOTHERAPY TREATMENT**	
Yes	36%
No	64%

### Physic and psychological impact on participant’s life

**Fig. 2:**
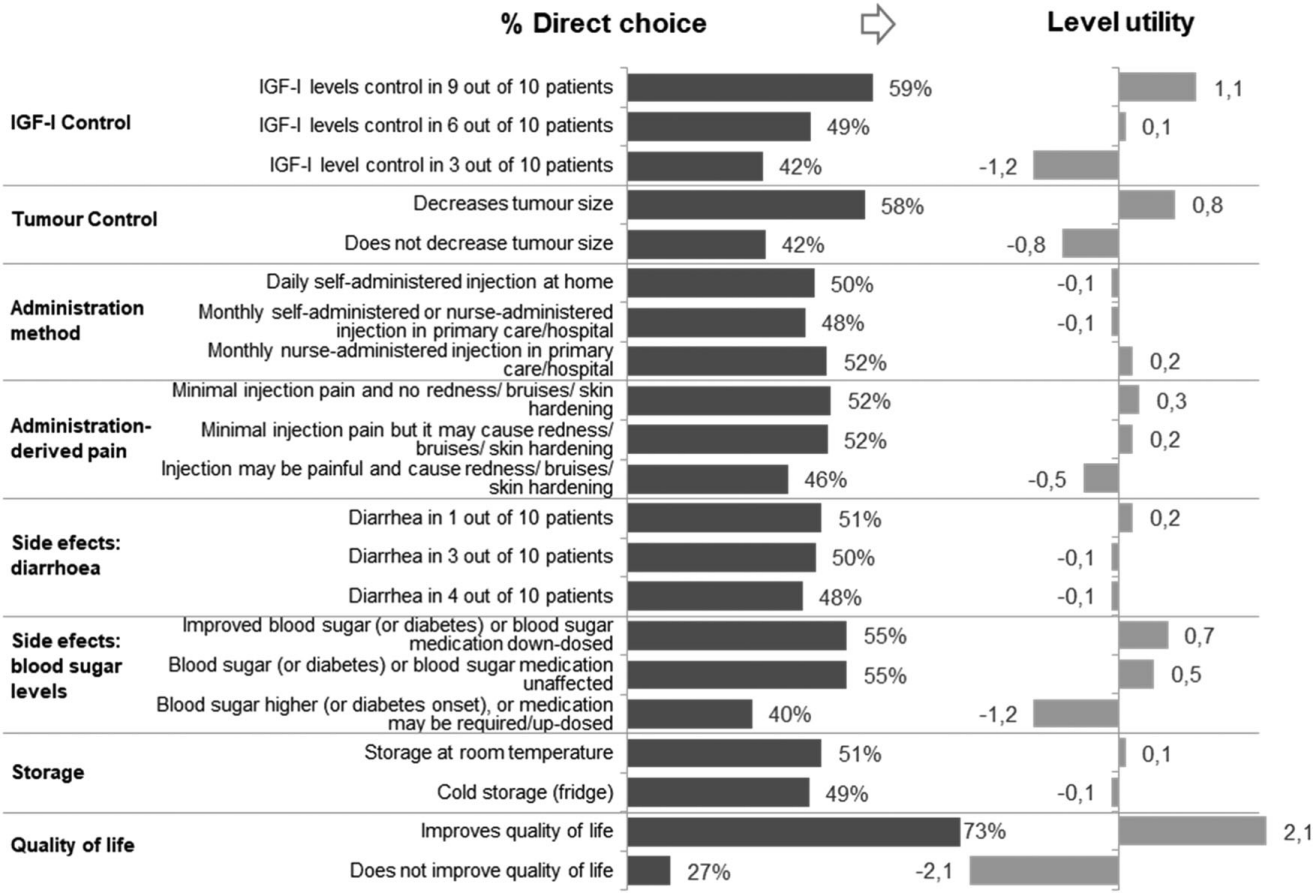
Level preference and utility. Utility quantitatively represents the relative value that the respondent attaches to each proposed level of each attribute, e.g. Quality of life is an attribute with two possible levels 'improves QoL' or 'does not improve QoL'. In this case, 'improves QoL' was deemed to be much more valuable than 'does not improve QoL' by patients. Partial utilities only make sense within the same attribute and depend on the number of proposed levels, i.e. they are relative values. The first column (% Direct choice) shows the percentage of times that each level was chosen, considering that all levels are supposed to be represented an equal number of times in the DCE. This is transformed into the level utility shown in the second column (Level utility). The relative difference in utility between 2 levels of the same attribute represents the strength of the preference for a particular level over the other

Figure 2 shows the preferences or utilities of acromegaly treatments for patients.

**Fig. 3:**
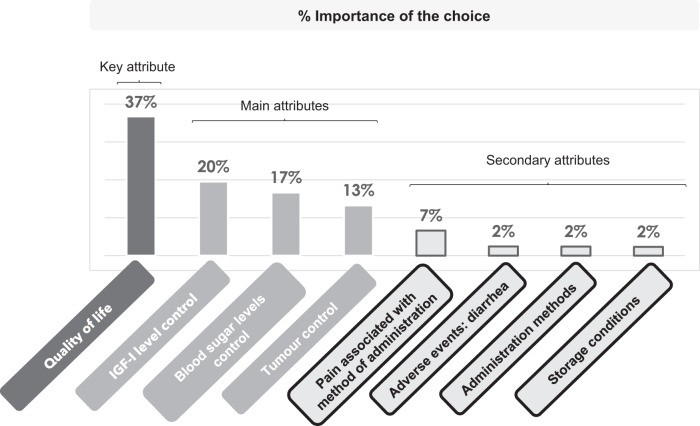
Utilities summary

Although IGF-I control was one of the most important attributes according to participants, together with blood sugar control and tumour control, QoL stood out as the most important attribute (Fig. 3).

The pain associated with treatment administration was one of the attributes that provided less utility to the treatment, although injection pain was more important than redness, bruises, or hardening of the skin. Diarrhoea and storage conditions were the last two attributes that formed the group of secondary attributes that define the choice of treatment when all other utilities are equal.

A subanalysis of utility was carried out according to patient characteristics in order to explore factors that may influence preference towards injectable treatments. No significant differences were found between male and female patients. The time since the start of treatment seems to have an impact on the importance of the blood sugar impact of medication. Namely, patients that had been on treatment for more than 8 years at the time of the study had a more marked preference for treatments that lowered or at least did not increase blood sugar levels compared to treatments that increased blood sugar levels (Supplementary Material Fig. S1). This did not differ significantly between diabetic and non-diabetic patients (Supplementary Material Fig. S2). Patients that were on first-line treatment preferred monthly administration by a nurse at the healthcare centre, while patients that had been under more than one treatment slightly preferred daily self-administration over the other options (Supplementary Material Fig. S3).

### Optimal treatment and share of preference for existing treatments

Considering respondent preferences, the characteristics of the ideal treatment were listed (Table 4).

**Table 4:** Ideal treatment according to patient preferences

IGF-I level control	IGF-I level control in nine out of ten patients
**Tumour control**	Reduces tumour size
**Administration methods**	Monthly nurse-administered injections in primary care/hospital
**Pain associated with the administration method**	Minimal injection pain and no redness/ bruising/skin hardening
**Adverse events, diarrhoea**	Diarrhoea in 1 out of 10 patients
**Blood sugar control**	Improved blood sugar (or diabetes) or blood-sugar medication down-dosed
**Storage conditions**	Storage at room temperature
**Quality of life**	Improves quality of life

*IGF-I* insulin-like growth factor I

**Fig. 4:**
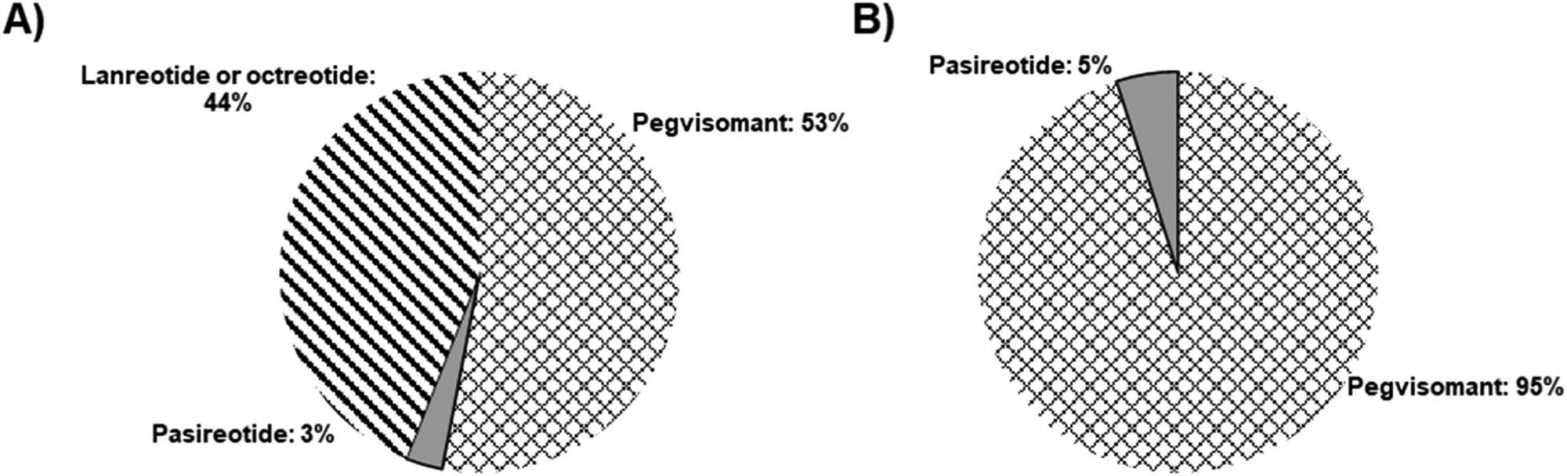
Share of preference of the different existing acromegaly treatments. Preference share was estimated on the basis of the utility of the attributes and levels, i.e. the treatment that was associated to the most preferred levels of each attribute obtained a higher percentage of share. **A** represents shared preferences among all existing treatments indicated for acromegaly, while **B** represents preference share between the two drugs available for second-line treatment

Following this, an analysis was performed where the partial preferences of the various attributes and levels were combined to estimate the share of preference for the existing acromegaly injectable drugs (Supplementary Table S2): first-generation somatostatin analogues (lanreotide or octreotide), pasireotide and pegvisomant. Figure 4 illustrates how the combination of the preference attributed to each treatment characteristic combines into real existing treatments. Pegvisomant would be the most preferred treatment according to patient preference, with a share of preference similar to the 1G-SRLs, with pasireotide taking a minimal share of preference (Fig. 4A). If we consider the two treatments that are given in the second line, pasireotide and pegvisomant, the difference in share is more evident (Fig. 4B).

In a further analysis, some of the attribute levels were modified hypothetically to test the impact they would have on patient preferences for treatment, efficacy in controlling IGF-I levels, tumour reduction, blood sugar level involvement, and storage temperature. Results are reported in Supplementary Material Fig. S4.

## Discussion

This study aimed to assess patient preferences for the attributes of acromegaly pharmacologic treatments in Spain. Results show that QoL was the attribute with the highest level of utility, and, therefore, the one that patients believed was most important, followed by IGF-I levels, blood sugar levels and tumour control.

Recent work found that there are currently 33 methods that have been used to assess patient preferences for treatment [34], and DCE is the most used in healthcare studies [35]. It assumes that the utility of a certain intervention is determined by different characteristics, called attributes, that describe it. Each attribute has different levels. Thus, statistical methods are used to combine the attribute levels to generate a series of independent choice tasks in which the participant has to trade-off the best alternative among two or three options [36]. The choices over a number of alternatives can then be analysed to calculate the relative importance of the attributes [36]. It is assumed that respondents take into account all information provided and then select the alternative which provides the highest utility to them. Changes in the attribute levels can alter the preferred choice alternative of participants [37]. Besides, it assumes that the participant choice behaviour is probabilistic rather than deterministic [36, 38]. DCE is particularly suited to deal with situations where changes are multi-dimensional, and trade-offs between the dimensions are of particular interest, because of their ability to separately identify the value of individual attributes when given in combination with one another. The main disadvantage lies in the cognitive difficulty associated with multiple complex choices when many attributes and levels are considered at once [39].

Among the many impairments in day-to-day life caused by disease and comorbidities, acromegaly patients must face an important reduction in QoL [27, 40–42]. Biochemical control does not ensure complete remission of symptoms and improvement in QoL; [3, 30] therefore, to optimise treatment, patient preferences, an important factor in disease management that has been largely ignored until recently [29], should be considered [27, 43]. Using the clinical decision support tool, Acromegaly Disease Activity Tool (ACRODAT^®^), which assesses five parameters: IGF-I levels, tumour status, comorbid conditions, signs/symptoms and QoL, the AcroVoice study found that these parameters were important to patients. Interestingly, the authors found that patient-centred parameters were more important for patients than IGF-I levels and tumour status [3]. QoL ranked high in patient preference for health status, but was not the most significant preference when changing treatment [3]. A possible reason is the quantitative character of IGF-I, which is easier to measure than QoL. In general, patients weighted patient-centred parameters more than clinicians [3].

Our results suggest that patients are aware of their biochemical control parameters, these being objective, easy-to-follow, quantitative measures. Blood sugar level control and tumour size influence the patient’s choice of treatment, and notably, patients gave more importance to having their blood sugar levels controlled than their tumour size. This may reflect their awareness of the risks related to their treatment and/or of diabetes as a possible complication of their disease. Their concern was for these levels to decrease or stabilise, with no preference. A group subanalysis has shown that this preference was irrespective of diabetes diagnosis or high blood sugar levels.

In terms of method of administration, respondent preferences for a daily or monthly regimen were equal, which may indicate that there is room for personalised treatment. Surprisingly, respondents preferred the injection to be administered at the primary care centre or the hospital by a nurse, rather than self-administration at home. Although this may be seen as a loss of autonomy, this interpretation should be viewed with caution, since the answers were very polarised. Respondents receiving their first treatment mainly preferred monthly injection by a healthcare provider, which may reflect their insecurity or their lack of experience with other treatment options, while more experienced patients (>1 treatment, possibly using pegvisomant) showed no difference in preference between healthcare personnel or self-administration (Supplementary Fig. S3). This suggests that patients who are new to treatment prefer direct supervision by a healthcare professional, while more experienced patients value more their independence, although individual preference varies [44]. Diarrhoea was traded-off as an acceptable side effect according to the survey, even though in the qualitative phase of the study, it was deemed to be a relevant side effect.

The current treatment algorithms for acromegaly are based on a 'trial-and-error' approach, with additional treatment options provided when the disease is not controlled [45]. In the context of personalised medicine, the inclusion of patient preferences could be beneficial. Shared decisions increase patient adherence to treatment and, therefore, effectivity [46]. Patients are informed or willing to be informed, and are aware and eager to take control of their disease and their treatment [47]. However, their opinion is usually disregarded.

This study shows that the preference of this cohort for pegvisomant and lanreotide/octreotide is mainly driven by its effect on IGF-I level control, sugar blood levels and the neutral effect on tumour volume. Pegvisomant was strongly preferred as a second-line treatment.

The limitations of the study are those inherent to the study design: as a survey, it reflects only the opinion of a limited number of patients. To avoid conditioning this opinion from the beginning, we decided to elicit relevant treatment attributes directly from the patients by means of two group interviews, with nine patients in total, carried out in different cities to exclude possible regional bias. This implies the risk that some of the relevant attributes may have remained outside the analysis; however, we reviewed a posteriori of the available literature on acromegaly patient preferences, and none of the previously reported relevant attributes are absent from our study [3, 44, 48]. Furthermore, the study investigators reviewed and validated the attributes to ensure appropriateness. Despite ensuring that the survey language was understandable to patients by validating the final questionnaire with patients from the Spanish Patient-Advocacy Group, the dynamics of the DCE may not have been entirely clear to all patients, since we obtained a relatively low rate of response to the questionnaire (62.62%). The exercise required a certain degree of concentration, and its digital presentation may also have been a barrier to patients less familiar with digital applications. The fact that the questionnaire was self-administered without either investigator or patient-advocacy group support may have prevented more patients from completing the questionnaire. However, this also ensures that results reflect the patient’s unbiased opinions. The main limitation of this study was the fact that participants were mainly recruited from the advocacy group, which may have resulted in a study population with more information, motivation, and awareness than the general acromegaly population. To offset this, the study investigators also recruited some study participants at their hospital.

Finally, data were collected between September 2020 and March 2021. Although the COVID-19 alert state was over by then in Spain, life was different from what patients had known before, and this could have affected their priorities. This study applies to a specific period and may not be relatable to a subsequent “normal-life” timeframe. However, COVID-19 is likely to persist in the near future, and this supports the long-term relevance of our study.

Although acromegaly patients show a high degree of awareness of the importance of IGF-I levels and tumour size control, our results show the importance they give to QoL. Moreover, patients showed great concern about glycaemic level alteration, implying they are informed of treatment side effects and have a comprehensive understanding of their disease, attaching great importance to both disease control and comorbidity risk. Thus, patient opinions should be taken in great consideration when prescribing treatment. Patient empowerment may greatly facilitate disease control.

QoL greatly influences patient treatment preferences. Since acromegaly patients have been shown to be well-informed and aware of their disease, treatment choices should always be shared with patients.

### Supplementary information

Supplementary Material
