# Supplementary material for: Preference of acromegaly patients for treatment attributes in Spain
Source: Endocrine. 2023 Jul 28;82(2):379–89. doi: 10.1007/s12020-023-03462-z (PMC10543785; doi:10.1007/s12020-023-03462-z)
Supplement: Supplementary file 1 — Supplementary Material [file 12020_2023_3462_MOESM1_ESM.docx]

SUPPLEMENTARY MATERIAL

**Table S1**. Description of levels and attributes.

| **Levels** | **Attribute 1: IGF-I level control** | **Treatment correspondence** |
| --- | --- | --- |
| **1** | IGF-I levels control in 9 out of 10 patients | Pegvisomant |
| **2** | IGF-I levels control in 6 out of 10 patients | Octreotide/Lanreotide |
| **3** | IGF-I level control in 3 out of 10 patients | Pasireotide |
| **Levels** | **Attribute 2: Tumour control** |  |
| **1** | Reduces tumour size | Octreotide/Lanreotide/Pasireotide |
| **2** | Does not reduce tumour size | Pegvisomant |
| **Levels** | **Attribute 3: Administration methods** |  |
| **1** | Daily self-administered injection at home | Pegvisomant |
| **2** | Monthly self-administered or nurse-administered injection in primary care/hospital | Lanreotide |
| **3** | Monthly nurse-administered injection in primary care/hospital | Octreotide/Pasireotide |
| **Levels** | **Attribute 4: Pain associated to the administration method** |  |
| **1** | Minimal injection pain and no redness/ bruising/ skin hardening | All treatments may cause pain or redness/bruises of the skin |
| **2** | Minimal injection pain but may cause redness/ bruising/ skin hardening |  |
| **3** | Injection may be painful and cause redness/ bruising/ skin hardening |  |
| **Levels** | **Attribute 5: Adverse events: diarrhoea** |  |
| **1** | Diarrhoea in 1 out of 10 patients | Pegvisomant |
| **2** | Diarrhoea in 3 out of 10 patients | Pasireotide |
| **3** | Diarrhoea in 4 out of 10 patients | Lanreotide/Octreotide |
| **Levels** | **Attribute 6: Blood sugar levels control in blood** |  |
| **1** | Improved blood sugar (or diabetes), or blood sugar medication down-dosed | Pegvisomant |
| **2** | Blood sugar (or diabetes) or blood sugar medication unaffected | Octreotide/Lanreotide |
| **3** | Blood sugar higher (or diabetes onset), or blood sugar medication may be required/up-dosed | Pasireotide |
| **Levels** | **Attribute 7: Storage conditions** |  |
| **1** | Storage at room temperature | Pegvisomant |
| **2** | Cold storage (fridge) | Lanreotide/Octreotide/Pasireotide |
| **Levels** | **Attribute 8: Quality of life** |  |
| **1** | Improves quality of life | All treatments may improve the QoL |
| **2** | Does not improve quality of life |  |

IGF-I levels 1,2 and 3 correspond to the efficacy reported for pegvisomant, lanreotide/octreotide and pasireotide, respectively. Level 1 of Tumour control correspond to the SSA analogues and level 2 to pegvisomant. Level 1, 2 and 3 of the administration methods’ attribute correspond to pegvisomant, lanreotide and octreotide/pasireotide, respectively. Diarrhoea attribute’s level 1, 2 and 3 correspond to pegvisomant, pasireotide and lanreotide/octreotide, respectively. Attribute 4 and 8 do not correspond to any treatment

Figure S1: Subgroup analysis of utility level of attributes and levels, depending on years on treatment


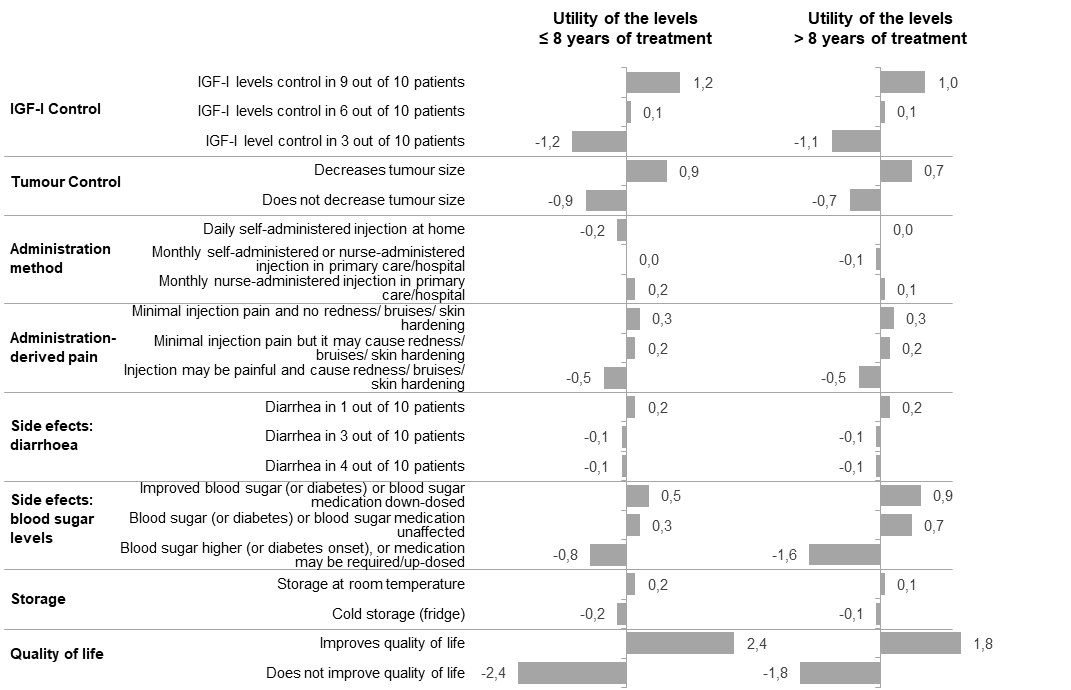


**Figure S2**: Subgroup analysis of utility level of attributes and levels, depending on diabetes diagnosis


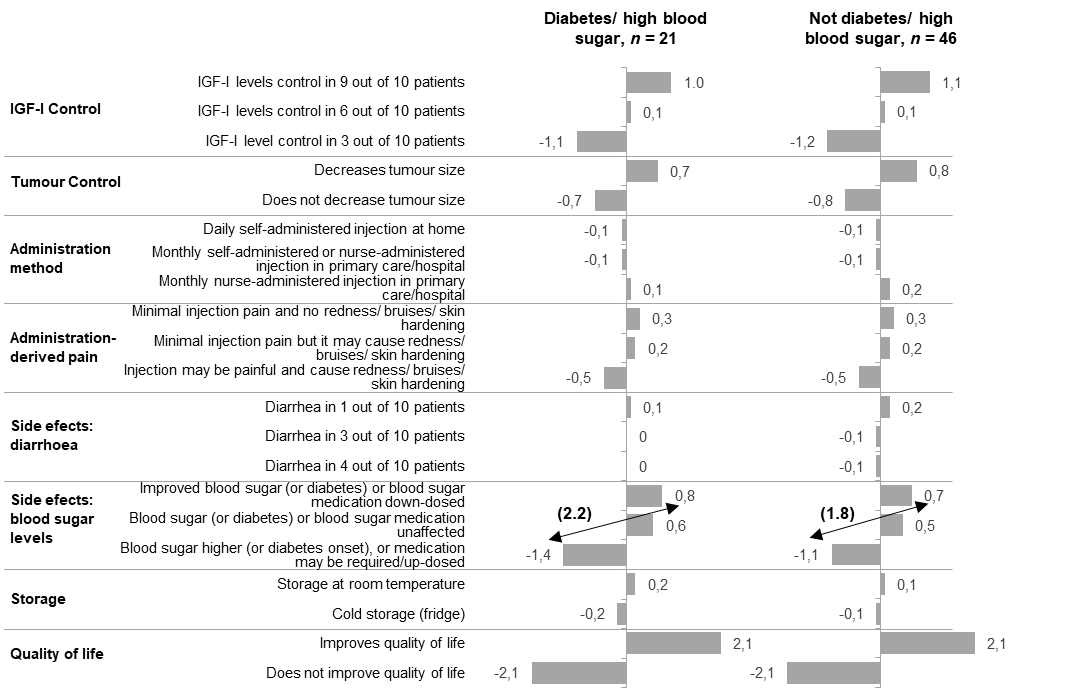


Arrows represent the absolute difference between the improvement of blood sugar levels or diabetes, or decrease of the medication and the rise in the levels or the need of medication.

**Figure S3**: Subgroup analysis of utility level of attributes and levels, depending on current line of treatment


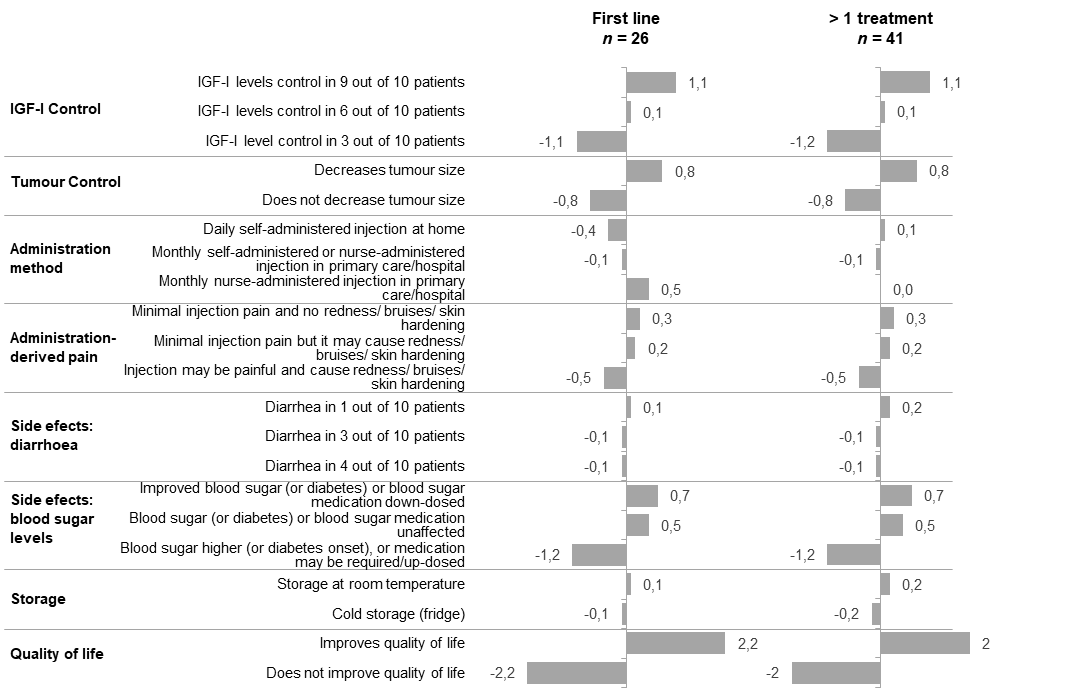


Table S2. Level of attributes for the three existing acromegaly treatments.

|  | **lanreotide or octreotide** | **pasireotide** | **pegvisomant** |
| --- | --- | --- | --- |
| **IGF-I level control** | 6 out of 10 patients | 3 out of 10 patients | 9 out of 10 patients |
| **Tumour control** | Reduces tumour size | Reduces tumour size | Does not reduce tumour size |
| **Administration methods** | Monthly self-administered or primary care/hospital nurse-administered injection* | Monthly nurse-administered injection in primary care/hospital | Daily self-administered injection at home |
| **Pain associated with the administration method** | Minimal injection pain and no redness/ bruising/ skin hardening | Minimal injection pain and no redness/ bruising/ skin hardening | Minimal injection pain and no redness/ bruising/ skin hardening |
| **Adverse events, diarrhoea** | Diarrhoea in 4 out of 10 patients | Diarrhoea in 3 out of 10 patients | Diarrhoea in 1 out of 10 patients |
| **Blood sugar control** | Blood sugar (or diabetes) or blood sugar medication unaffected | Blood sugar higher (or diabetes onset), blood sugar medication may be required/up-dosed | Improved blood sugar (or diabetes) improved, or blood sugar medication down-dosed |
| **Storage conditions** | Cold storage (fridge) | Cold storage (fridge) | Storage at room temperature |
| **Quality of life** | Improved quality of life | Improved quality of life | Improved quality of life |

* To estimate patient preference for octreotide and lanreotide, treatments were unified; however, octreotide can only be nurse-administered in primary care/hospital .

**4**
